# Establishment of a dental license regulation authority is required in Korea: results of the Delphi technique

**DOI:** 10.3352/jeehp.2017.14.11

**Published:** 2017-05-29

**Authors:** Jin-Woo Choi, Kack-Kyun Kim, Jihyun Lee, Dong-Ju Choi, Kyung-Nyun Kim

**Affiliations:** 1 College of Dentistry, Dankook University, Cheonan, Korea; 2 School of Dentistry, Seoul National University, Seoul, Korea; 3 Life Dental Clinic, Seoul, Korea; 4 College of Dentistry, Gangneung-Wonju National University, Gangneung, Korea; Hallym University, Korea

**Keywords:** Delphi technique, Dental licensure, Quality control

## Abstract

**Purpose:**

In addition to dental education, a system for the evaluation and management of dental licensing and certification is required to meet the growing societal demand for more competent dentists. In this study, the Delphi technique was used to gather opinions from a variety of professionals on the problems of and remedies for the dental license management system in Korea.

**Methods:**

Delphi surveys were conducted from April 2016 to October 2016 in South Korea. A variety of dental professionals were included and categorized into 3 groups according to their expertise as follows: the basic dentistry group, the clinical dentistry group, and the policy group. The Delphi technique was conducted in 3 rounds of e-mail surveys, each with different questions that probed with increasing depth on the dental license management system. In each successive round, the responses were categorized, scored on a Likert scale, and statistically analyzed.

**Results:**

After categorizing the results of the first survey and ranking the results of the second survey using the Delphi technique, regulation by a licensing authority was found to be the most critical issue. This was followed by the license renewal system, continuing education, a tiered licensure system, improvement of foreign license approval, and utilization of retirees, in decreasing order of importance. The third Delphi survey showed a similar ranking, with regulation by a licensing authority being the major concern. Opinions regarding the dental license management system were provided as open-ended responses. The responses of the 3 groups showed statistically significant differences in the scores for the issue of regulation by a licensing authority. After re-grouping into the dentistry group and the policy group, the issue received a significantly higher score in the dentistry group.

**Conclusion:**

The quality of dental treatment should be managed to protect patients and dental professionals. For this purpose, the establishment of an independent license regulation authority along with legislative changes is required.

## Introduction

Changes in the population and disease structure and the development of new medical technology have dramatically transformed the oral healthcare environment. This, in turn, has led to changes in medical policy and has increased the required expertise for dentists. Although it is essential that dentists receive formal dentistry education at a university in order to competently satisfy societal demands, a system for the evaluation and management of licensing and certification is also of utmost importance. Furthermore, a systematic management policy for dental licensing must be established to protect citizens and improve oral health in an age where healthcare personnel continue to move across borders. In this study, the Delphi technique was used to gather opinions from a variety of professionals, such as local practitioners, dental school professors, policy makers, and consumer representatives in order to gain an understanding of the problems of and remedies for the dental license management system.

## Methods

### Study design

A cross-sectional study design was used.

### Materials and subjects

When using the Delphi technique, it is important to carefully consider the participants’ representativeness, their professional knowledge, and the integrity of participation and answers; for this reason, the study sample was organized into the groups listed in Table 1. The study sample consisted of 21 participants and included professionals with experience at the Korea Health Personnel Licensing Examination Institute, local practitioners, basic dentistry professionals, clinical dentistry professionals, policy makers, medical experts, and humanities and social sciences-related dentistry professionals. The participants were grouped according to expertise, with 6 people in the basic dentistry group, 10 people in the clinical dentistry group, and 5 people in the policy group.

The selection of an appropriate number of rounds of surveying is a critical part of reaching a consensus using the Delphi technique. Three rounds of surveys are typically recommended to reach an agreement among experts, since the variation in responses is the greatest during the first 2 rounds. In this study, we followed this recommendation and conducted 3 rounds of e-mail surveys.

### Questionnaire

The first Delphi survey asked the participants about issues regarding the current dental license, the license management system, and potential improvements to the system. Open-ended questions were used to obtain a wide range of responses and opinions from the expert groups. Existing competency models of both the Korean Institute of Dental Education and Evaluation and the International Society of Dental Regulators were provided to encourage thinking about issues regarding dentist competencies [1,2] (Appendix 1).

The second Delphi survey asked the respondents to rate the importance of 6 issues categorized from the results of the first survey on a 5-point Likert scale (from 1 to 5). An open-ended question asking for additional opinions on dental license policy and management was also included (Appendix 2).

The third Delphi survey compiled the responses from the second survey, compared them to each individual expert’s responses, and asked the expert to provide modified responses as needed. Additionally, the respondents were asked to reevaluate the importance of the 6 issues (Appendix 3).

### Statistics

All 21 of the participants responded to all 3 surveys. Similar opinions acquired from the open-ended responses in the first survey were categorized into 6 issues according to the consensus of 4 researchers. The importance of each issue was determined by averaging the Likert scale scores from the second survey. The issues were ranked by weighting each averaged score (from rank 1 [5 points] to rank 5 [1 point]), multiplying the rank by the number of responses, and converting the product into a score out of a maximum of 100 points. The same method was used for the third survey, and its results were compared to those of the second survey. The non-parametric Kruskal-Wallis test was used to verify the differences in scores among expert groups for each issue. The Mann-Whitney test was then conducted on significant results from the Kruskal-Wallis test. SPSS ver. 12.0 (SPSS Inc., Chicago, IL, USA) was used for statistical analysis, with P< 0.05 considered statistically significant.

### Ethical approval

This research (registration number S-020160015) was authorized by the institutional review board at the Seoul National University School of Dentistry.

## Results

### First survey results

The categorized issues formed from the open-ended responses in the first survey were regulation by a licensing authority, continuing education, the license renewal system, a tiered licensure system, improvement of foreign license approval, and utilization of retirees. The number of responses for each issue is presented in Table 2. Raw data were available from Supplement 1.

### Second survey results

**Fig. 1.**
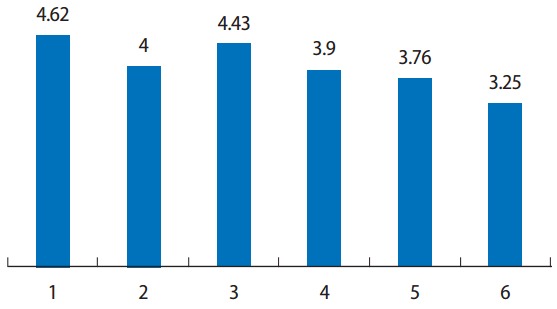
Importance of potential improvements for license management (second round). 1, regulation by licensing authority; 2, continuing education; 3, license renewal system; 4, leveled license system; 5, improvement of foreign license approval; 6, utilization of retirees.

**Fig. 2.**
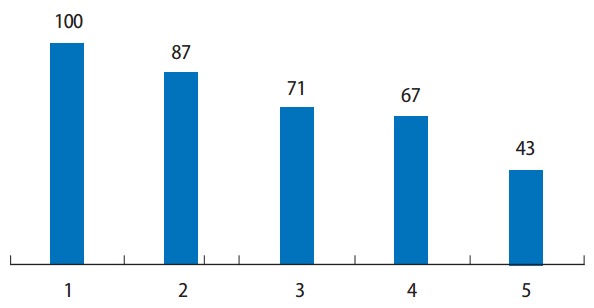
Priority ranking of potential improvements for license management (second round). 1, regulation by licensing authority; 2, continuing education; 3, license renewal system; 4, leveled license system; 5, improvement of foreign license approval; 6, utilization of retirees.

According to the second Delphi survey, the issues were ranked in descending order as follows: regulation by a licensing authority, the license renewal system, continuing education, a tiered licensure system, improvement of foreign license approval, and utilization of retirees (Fig. 1). Regulation by a licensing authority was the most important issue, even in the rankings for potential improvements for the license management system, followed by continuing education, the license renewal system, a tiered licensure system, and improvement of foreign license approval (Fig. 2).

### Third survey results

The third Delphi survey also showed that regulation by a licensing authority was considered the most important issue, and the rankings of the other issues did not differ significantly from those of the second survey (Table 3). Although the priority ranking did not change significantly, continuing education moved to a higher rank than a tiered licensure system (Table 4).

After 3 rounds of Delphi surveys, we found in all surveys that the participants identified regulation by a licensing authority as the most important area of concern, followed by the license renewal system, continuing education, and a tiered licensure system. The following conclusions were made based on the open-ended responses. First, many experts believed that the license regulation authority should be independent. Second, some experts voiced the opinion that clinical education of undergraduate students in dental school could be strengthened by implementing a tiered licensure system. Third, a majority of the experts agreed that the quality of continuing education required reform and acknowledged that such plans would require more investment. Fourth, concern was raised about the need for stringent restriction and mutual authentication of foreign licenses.

### Comparisons among expert groups

The results of the Delphi technique can change with the selection of the experts and the constituent ratio. Therefore, differences in responses among expert groups were analyzed to evaluate whether the research results could be standardized, irrespective of the expert group. The 21 experts who participated in this research were categorized into 3 groups: the clinical dentistry group, the basic dentistry group, and the policy group. In order to check whether there was a statistical difference in the priority ranking among the groups, the nonparametric Kruskal-Wallis test was conducted. The test showed that there was a statistically significant difference among the responses of the groups for the issue of regulation by a licensing authority (P<0.05). In order to verify these differences, the Mann-Whitney test was conducted. However, it yielded no significant results (P> .0167) (Tables 5 and 6). Furthermore, when the basic dentistry and clinical dentistry groups were combined into a single dentistry group, a significantly higher score was observed for the issue of regulation by a licensing authority than was observed in the policy group (Table 7). No significant differences were observed for the other issues (Table 5).

## Discussion

A license management system typically consists of granting, registration, and renewal of licenses; educational programs to ensure professionalism; and procedures through which public complaints regarding medical practices can be resolved [3]. In Korea, a dental license can currently be obtained by graduating from a dental university or a graduate school of dentistry that is approved by the Ministry of Health and Welfare and then passing a Korean dental licensing examination in the form of a paper-and-pencil test. Once the license is obtained, it can be re-issued every 3 years by completing a certain amount of supplementary education. However, there is no regulatory system other than the requirement to complete 8 hours of supplementary education annually. It is, therefore, impossible to verify a dentist’s qualifications for opening a local clinic or renewing a certification, or to take disciplinary action for the improper provision of dental treatment. It is unwise to have a system that relies solely on individual dentists, which may lead to an environment in which the quality of dental treatment is poorly managed. As the speed of technological advancement increases along with the movement of medical personnel overseas, the need to reform the outdated license management system grows.

In this study, we used the Delphi technique in order to gain a better understanding about reforms to the license management system. The results showed that experts from a variety of fields believed that establishing an independent license regulation authority is of the utmost importance. Experts within the field of dentistry appeared to believe this much more strongly than the policy experts. The reason for this difference in opinion is not clear; however, it is suspected that the experts who work in dental hospitals are more likely to experience unprofessional behavior by dentists, and, thus, they feel more strongly about the need for a regulation authority. In other countries, such as the United States, Australia, the United Kingdom, Germany, France, the Netherlands, Romania, Cyprus, Czech Republic, Denmark, Switzerland, Sweden, and Poland, licenses are managed independently and dentists can be warned, fined, or even have their licenses suspended if they exhibit improper behavior during the provision of dental treatment [4,5]. The establishment of an independent license regulation authority can ensure professionalism and protect that autonomy of dentists, provide citizens with quality dental services, and increase trust between dentists and patients. Despite reports on the need for a license regulation authority [3], current medical law does not allow a regulatory authority to operate. Thus, any such change to the current system must be accompanied by legislative reforms.

In addition to the establishment of a license regulation authority, other policies, such as a license renewal system, the strengthening of continuing education, and a tiered licensure system, were deemed necessary by the experts. A tiered licensure system is also a method in which the quality of dental services is managed, and in the United States, Germany, Japan, and China, there are tiered licensure systems for independent practice and running a hospital [4,6,7]. In Japan, a license for independent practice is granted after at least 1 year of clinical training at a hospital certified by the Ministry of Health, Labor, and Welfare, while in China, a license to run a hospital is granted by passing a dental skills evaluation or by proving at least 5 years of clinical experience in the same subject. Germany has a claim for health insurance that it uses in place of a license to run a hospital, which requires at least 1 year of experience as an assistant dentist. Of the countries considered in this research, only the United States requires a separate practical test to obtain a license for independent practice. However, it should be noted that the United States requires the practical test to be conducted by a state-level license management institution rather than a federal institution. Upon researching many different countries, we have found that while many countries have a separate hospital license system, there are no additional exams required by the government other than the national examination, and evidence of treatment experience is often required. Therefore, even if a hospital license system is implemented, it seems wiser to have a variety of evaluation methods, such as clinical education, testimonies of treated patients, and portfolios, rather than a simple examination to pass.

## Figures and Tables

**Table 1.** Delphi survey participants and background

No.	Name	Affiliation	Special field	Representative area
1	OO, Lee	College of Dentistry (A)	National license examination	Basic and social dentistry
2	OO, Seo	College of Dentistry (B)	Accreditation of dental schools	Basic and social dentistry
3	OO, Park	College of Dentistry (C)	Basic dentistry	Basic and social dentistry
4	OO, hung	College of Dentistry (D)	Social dentistry	Basic and social dentistry
5	OO, Kim	College of Dentistry (C)	Social dentistry	Basic and social dentistry
6	OO, Kang	College of Dentistry (E)	Social dentistry	Basic and social dentistry
7	OO, Cho	Dental clinic (F)	Local clinic	Clinical dentistry
8	OO, Lee	Dental clinic (G)	Local clinic	Clinical dentistry
9	OO, Chung	Dental clinic (H)	Local clinic	Clinical dentistry
10	OO, Chung	Dental clinic (I)	Local clinic	Clinical dentistry
11	OO, Kim	Dental clinic (J)	Local clinic	Clinical dentistry
12	OO, Kim	College of Dentistry (K)	Clinical dentistry	Clinical dentistry
13	O, Chung	College of Dentistry (L)	Basic dentistry	Clinical dentistry
14	OO, Shin	College of Dentistry (K)	National license examination	Clinical dentistry
15	OO, Shim	College of Dentistry (B)	National license examination	Clinical dentistry
16	OO, Park	College of Dentistry (C)	National license examination	Clinical dentistry
17	OO, Kim	Ministry of Health and Welfare	Dental policy	Policy related group
18	OO, Byun	Korean Dental Association	Dental policy	Policy related group
19	OO, Yang	Attorney's office (M)	Dental policy	Policy related group
20	OO, Lee	College of Medicine (N)	Medical doctor	Policy related group
21	OO, Lee	College of Medicine (O)	Consumer	Policy related group

**Table 2.** First Delphi survey results

Category	Number (%)
Regulation by licensing authority	10 (29)
Continuing education	9 (26)
License renewal system	8 (23)
Leveled license system	5 (14)
Improvement of foreign license approval	2 (6)
Utilization of retirees	1 (3)
Total 6 categories	35 (100)

**Table 3.** Importance level evaluation results from the second and third Delphi surveys

Evaluated option	Second response results	Third response results
Regulation by licensing authority	4.62	4.62
License renewal system	4.43	4.33
Continuing education	4.00	4.00
Leveled license system	3.90	3.81
Improvement of foreign license approval	3.76	3.71
Utilization of retirees	3.25	3.19

Remarks: no clear changes in responses.

**Table 4.** Priority ranking results from the second and third Delphi surveys

Priority ranking			
Second response results		Third response results	
Regulation by licensing authority	100	Regulation by licensing authority	100
License renewal system	87	License renewal system	84
Leveled license system	71	Continuing education	70
Continuing education	67	Leveled license system	67
Improvement of foreign license approval	43	Improvement of foreign license approval	28

Remarks: changes in the third and fourth ranks.

**Table 5.** Comparison of average importance levels among expert groups

Evaluated option	Average	Standard deviation	P-value
Regulation by licensing authority			0.03
Basic dentistry group (6)	4.83	0.41	
Clinical dentistry group (10)	4.80	0.63	
Policy related group (5)	4.00	0.71	
Average	4.62	0.67	
Continuing education	0.07		
Basic dentistry group (6)	4.00	0.63	
Clinical dentistry group (10)	3.70	0.67	
Policy related group (5)	4.60	0.55	
Average	4.00	0.71	
License renewal system			0.64
Basic dentistry group (6)	4.33	0.82	
Clinical dentistry group (10)	4.20	0.92	
Policy related group (5)	4.60	0.89	
Average	4.33	0.86	
Leveled license system	0.94		
Basic dentistry group (6)	4.00	0.89	
Clinical dentistry group (10)	3.70	1.42	
Policy related group (5)	3.80	0.84	
Average	3.81	1.12	
Improvement of foreign license approval			0.71
Basic dentistry group (6)	3.67	0.82	
Clinical dentistry group (10)	3.80	0.79	
Policy related group (5)	3.60	0.89	
Average	3.71	0.78	
Utilization of retirees	0.50		
Basic dentistry group (6)	3.33	1.37	
Clinical dentistry group (10)	2.90	0.74	
Policy related group (5)	3.60	1.14	
Average	3.20	1.03	

a) Showed a statistically meaningful difference (Kruskal-Wallis test).

**Table 6.** Mann-Whitney U-test for the category ‘regulation by licensing authority

	Basic dentistry group		Clinical dentistry group	
	Clinical dentistry group	Policy related group	Basic dentistry group	Policy related group
Exact significance (P)	0.875	0.082	0.875	0.055

The P-value (0.0167; 0.05/3) was calculated with the Bonferroni correction.

**Table 7.** Difference in importance score between dentistry and policy related groups (Mann-Whitney U-test)

	Regulation by licensing authority^a)^	Continuing education	License renewal system	Leveled license system	Improvement of foreign license approval	Utilization of retirees
P-value	0.032	0.050	0.445	0.842	0.603	0.354

a) Showed a statistically significant difference.

## Supplementary materials

Supplement 1. Data file is available from https://dataverse.harvard.edu/dataset.xhtml?persistentId=doi:10.7910/DVN/SBLMI6

Supplement 2. Audio recording of the abstract.

## Appendix 1. First Delphi survey questionnaire

What do you consider to be problems and potential improvements for dental license policy and management? (Reference: Republic of Korea National Dentist Competency Model, International Society of Dental Regulators [ISDR] Dentist Competency Model)

<Reference> Core competency of dentists at a national level (Korean Institute of Dental Education and Evaluation, 2014)

1. Recognizing and abiding to social and ethical responsibilities as dental professionals

2. Self-development through recognizing the importance of active participation in life-long education

3. Ability to communicate effectively and cooperate with peers

4. Ability to acquire and apply dental knowledge and skills

5. Ability to diagnose patients by identifying their symptoms and medical histories to conduct proper tests

6. Ability to appropriately treat or prevent oral and facial diseases

7. Ability to handle emergencies in a dental hospital

<Reference> ISDR

Proposed ISDR dentist competencies

1. Professionalism

2. Diagnosis & treatment

3. Communication & collaboration

4. Oral health promotion

5. Practice management

## Appendix 2. Second Delphi survey questionnaire

1. The following opinions were voiced regarding issues and potential improvements in the current dental license system and management.

1) Regulation by a licensing authority: dentist quality management through a license maintenance and management institution, use of self-discipline to manage unethical behavior and licenses, and strengthening of the license quality control system after granting licenses.

2) Continuing education: requirement for the management of education score (includes online education) and an obligation to participate in clinical treatment in addition to 8 hours of supplementary education annually, including an ethics education oath process.

3) License renewal system: the license renewal system must be strengthened (license can be maintained very easily now), reissuing of licenses after a certain period of time, signing of a pledge when reissuing a license and notice of confiscation upon the breaking of the pledge, the need to separate dental staff with a medical history, and implementation of a license registration policy.

4) Tiered licensure system: a tiered licensure system that can ensure an appropriate treatment capability is required, the dentist qualification test and hospital license test should be differentiated, hospital license should be only granted after a period of on-site experience, and differentiation between certification and license (separation of general dentistry, integrated dentistry, and other specializations) is required.

5) Improvement of foreign license approval: it is necessary to reform license standards for foreigners who graduated from dental universities abroad, such as from the United States, Japan, and Western Europe, including potential methods such as mutual recognition or disallowance of obtaining domestic licenses.

6) Utilization of retirees: a plan to use retired medical personnel is required.

2. Please check your level of agreement for each choice, and prioritize the options from 1 to 5

**Table:**

	Level of agreement					Priority
	Strongly disagree	Disagree	Neutral	Agree	Strongly agree	
Regulation by a licensing authority	1	2	3	4	5	
Continuing education	1	2	3	4	5	
License renewal system	1	2	3	4	5	
Tiered licensure system	1	2	3	4	5	
Improvement of foreign license approval	1	2	3	4	5	
Utilization of retirees	1	2	3	4	5	

3. Please state any additional areas of improvement and/or other opinions here

## Appendix 3. Third Delphi survey questionnaire

1. According to the results of the second Delphi survey, the level of agreement and priority ranking regarding problems of and potential improvements to the dentist license policy and management areas are as follows:

1, regulation by a licensing authority; 2, continuing education; 3, license renewal system; 4, tiered licensure system; 5, improvement of foreign license approval; 6, utilization of retirees.

1, regulation by a licensing authority; 2, continuing education; 3, license renewal system; 4, tiered licensure system; 5, improvement of foreign license approval.

2. Please check your level of agreement for each choice, and prioritize the options from 1 to 5

**Table:**

	Level of agreement					Priority
	Strongly disagree	Disagree	Neutral	Agree	Strongly agree	
Regulation by a licensing authority						
Your previous response	1	2	3	4	5√	1
Modified current response						
Continuing education						
Your previous response	1	2	3	4√	5	4
Modified current response						
License renewal system						
Your previous response	1	2	3√	4	5	3
Modified current response						
Tiered licensure system						
Your previous response	1	2√	3	4	5	2
Modified current response						
Improvement of foreign license approval						
Your previous response	1√	2	3	4	5	5
Modified current response						
Utilization of retirees						
Your previous response	1	2	3	4	5	
Modified current response						

3. Please state any other suggestions that you may have in the box below
